# Ultrasound-Assisted Wound Debridement for Diabetic Foot Ulcers: A Systematic Review and Meta-Analysis of Randomized Controlled Trials

**DOI:** 10.3390/biomedicines14040846

**Published:** 2026-04-08

**Authors:** Shasha Mei, Hua Chen, Jiezhi Dai

**Affiliations:** 1 Department of Nursing, Shanghai 6th Peoples Hospital, Shanghai Jiao Tong University, Shanghai 200240, China; mss1990@sjtu.edu.cn; 2 Department of Anesthesiology, Shanghai 6th Peoples Hospital, Shanghai Jiao Tong University, Shanghai 200240, China; 3 Department of Orthopedic Surgery, Shanghai 6th Peoples Hospital, Shanghai Jiao Tong University, Shanghai 200240, China

**Keywords:** diabetic foot ulcer, ultrasound-assisted wound debridement, wound healing, meta-analysis

## Abstract

**Background:** Diabetic foot ulcers (DFUs) represent a severe and costly complication of diabetes mellitus. This meta-analysis aims to compare the efficacy of ultrasound-assisted wound debridement (UAWD) and conventional debridement in promoting wound healing in patients with DFUs. **Methods:** A systematic literature search was conducted using PubMed, EMBASE, BIOSIS, Web of Science, and the Cochrane Library from inception to 31 October 2025. Randomized controlled trials (RCTs) that compared UAWD with a placebo or standard wound care in patients with DFUs were included. Primary outcomes were the healing rate, time to complete healing, and reduction in wound area. Results were expressed as odds ratios (ORs) or mean differences (MDs) with 95% confidence intervals (CIs). This study was registered on the PROSPERO platform (CRD420251229633). **Results:** Ten RCTs that involved 386 patients were included. The meta-analysis showed that the treatment group had a significantly higher complete wound healing rate compared with the control group (OR: 2.92; 95% CI: 1.82 to 4.70; *p* = 0.75; I^2^ = 0%). The rate of wound area reduction was also significantly greater in the treatment group (MD: 21.29%; 95% CI: 3.03 to 39.56; *p* = 0.003; I^2^ = 75%). Furthermore, the time to complete healing was significantly shorter in the treatment group (MD: −4.84 weeks; 95% CI: −8.65 to −1.03; I^2^ = 61%, *p* = 0.05). **Conclusions:** UAWD appears to be more effective than conventional debridement alone in improving healing rates and accelerating wound closure in diabetic foot ulcers. However, safety data were inadequately reported across most included studies, with adverse events poorly characterized. Future large-scale RCTs should prioritize rigorous adverse event reporting to establish both the efficacy and safety profile of this intervention.

## 1. Introduction

Diabetic foot ulcer (DFU) represents one of the most severe and costly complications of diabetes mellitus [1]. During their lifetime, 19–34% of individuals with diabetes may develop a DFU, with a substantial proportion requiring amputation of the affected limb [2,3]. Therefore, there is an urgent need for innovative and effective wound management strategies.

The current treatment guidelines for DFU recommend debridement, infection management, revascularization, and decompression to promote healing [4]. A critical element of wound management is debridement, which involves the removal of non-viable tissue from the wound bed to facilitate its replacement with healthy tissue and accelerate healing [5]. Debridement may be performed using mechanical, sharp/surgical, autolytic, enzymatic, or biological methods [6,7]. Among these, sharp or surgical debridement is recommended by the International Working Group on the Diabetic Foot as the preferred approach, given its rapidity, cost-effectiveness, and widespread availability for wound bed preparation [6].

Recently, ultrasound-assisted wound debridement (UAWD) has emerged as an innovative therapeutic modality with significant potential to transform conventional DFU management [8]. A case series by Campitiello et al. demonstrated that the application of an ultrasonic debridement system reduces debridement time, preserves viable tissue safely, and is associated with low complication rates in DFU surgery [9]. By utilizing ultrasonic energy, UAWD enables precise removal of necrotic tissue, disruption of biofilms, and reduction of microbial load within ulcer lesions, thereby potentially accelerating the healing process [10,11]. Furthermore, the acoustic energy delivered via ultrasonic waves is proposed to stimulate essential wound healing processes, including enhanced angiogenesis and accelerated formation of granulation tissue [12]. However, despite a compelling theoretical rationale and promising outcomes reported in preliminary studies, the scientific community remains cautious. Current evidence supporting the clinical efficacy of UAWD in DFU management remains fragmented and inconsistent.

Previous systematic reviews on ultrasound-assisted wound debridement for diabetic foot ulcers have been limited by incomplete search strategies. To address these gaps, the present study offers several methodological advancements: (1) restricting inclusion to RCTs, (2) limiting inclusion to English-language publications, and (3) providing an updated literature search. By addressing these limitations, we aim to provide a more robust and clinically interpretable synthesis of the evidence.

This study therefore aimed to synthesize available evidence from RCTs to comprehensively compare the efficacy and safety of UAWD versus conventional debridement in DFU management.

## 2. Methods

### 2.1. Search Strategy

This systematic review was conducted in accordance with the Preferred Reporting Items for Systematic Reviews and Meta-Analyses (PRISMA) guidelines. It was registered on the PROSPERO platform (CRD420251229633).

A comprehensive literature search was performed across multiple electronic databases, including PubMed, EMBASE, BIOSIS, Web of Science, and the Cochrane Library. The study was carried out from inception to 31 October 2025, with only English-language papers being considered. The search terms were as follows: (‘ultrasonic debridement’ or ‘ultrasound debridement’ or ‘sonic debridement’) AND (‘diabetic foot’, ‘diabetic wound’, or ‘DFU’). The detailed search strategies are described in Supplementary Materials File S1. In addition to the electronic database search, the reference lists of all identified articles were manually screened to capture any potentially eligible studies not retrieved through the initial search. Grey literature and clinical trial registries were also searched.

### 2.2. Inclusion and Exclusion Criteria

#### 2.2.1. Inclusion Criteria

The inclusion criteria were as follows: (1) type of studies: only RCTs evaluating the efficacy of UAWD for DFUs were included; (2) type of participants: patients were adults diagnosed with DFU, regardless of age, gender, or diabetes type; (3) type of intervention: patients in the treatment group underwent UAWD and those in the control group were treated with a placebo or standard wound care; (4) outcomes: wound healing rate, time to complete healing, percentage reduction in wound area, and adverse events. Standard wound care was defined as the routine clinical practice at the study institution, which could include surgical debridement, mechanical debridement, dressing changes, offloading, and infection control.

The exclusion criteria included: (1) reviews and case reports; (2) the data was incomplete.

#### 2.2.2. Data Extraction

Two investigators independently extracted the following data from each included study: first author, publication year, study design, country, sample size, treatment details (UAWD and control), treatment time, and outcomes. Any disagreements in the data extraction process were adjudicated by a third reviewer. Wound healing was defined as complete epithelialization of the DFUs. The healing rate was calculated (No. of healed patients/No. of patients per group × 100). Time to healing was defined as the time from the day of ulcer inclusion in the study to wound healing. The percentage of wound area reduction was calculated by comparing the wound area at week zero to the wound area at the end of treatment.

#### 2.2.3. Quality Assessment

The methodological quality of the included studies was evaluated with the Cochrane Risk of Bias Tool for Randomized Controlled Trials (RCTs). This assessment was conducted independently by two authors across seven domains: random sequence generation, allocation concealment, blinding of participants and personnel, blinding of outcome assessment, incomplete outcome data, selective reporting, and other bias. The quality of evidence was graded through the Grading of Recommendations Assessment Development and Evaluations (GRADE) approach. The GRADE system summarizes the evidence quality on a 4-point scale (very low to high) [13].

### 2.3. Statistical Analysis

All statistical analyses were performed using Review Manager 5.4.1 software and Stata 14.1 software. For continuous outcomes, the effect sizes were expressed as mean differences (MDs) with 95% confidence intervals (95% CIs). For dichotomous data, odds ratios (ORs) with 95% CIs were calculated. Multi-arm trials were handled using the control group splitting method (Cochrane Handbook) to avoid unit-of-analysis errors.

Given the anticipated clinical heterogeneity across studies (e.g., differences in ultrasound devices, treatment protocols, and follow-up durations), the random-effects model was used as the default approach for all meta-analyses to account for both within- and between-study variability. Statistical heterogeneity was assessed using the I^2^ statistic; however, model selection was not solely based on I^2^ thresholds but rather on clinical and methodological considerations. Sensitivity analyses were conducted to assess the robustness of the findings. Specifically, we compared the pooled effect sizes calculated using risk ratios (RRs) versus odds ratios (ORs). Additionally, we compared the results obtained from random-effects models with those from fixed-effect models. The influence of individual studies on the overall effect was evaluated using the leave-one-out method. Publication bias was assessed using funnel plots, Begg’s test, and Egger’s test. A two-sided *p*-value of less than 0.05 was considered statistically significant for all analyses.

## 3. Results

### 3.1. Study Selection and Characteristics

**Figure 1:**
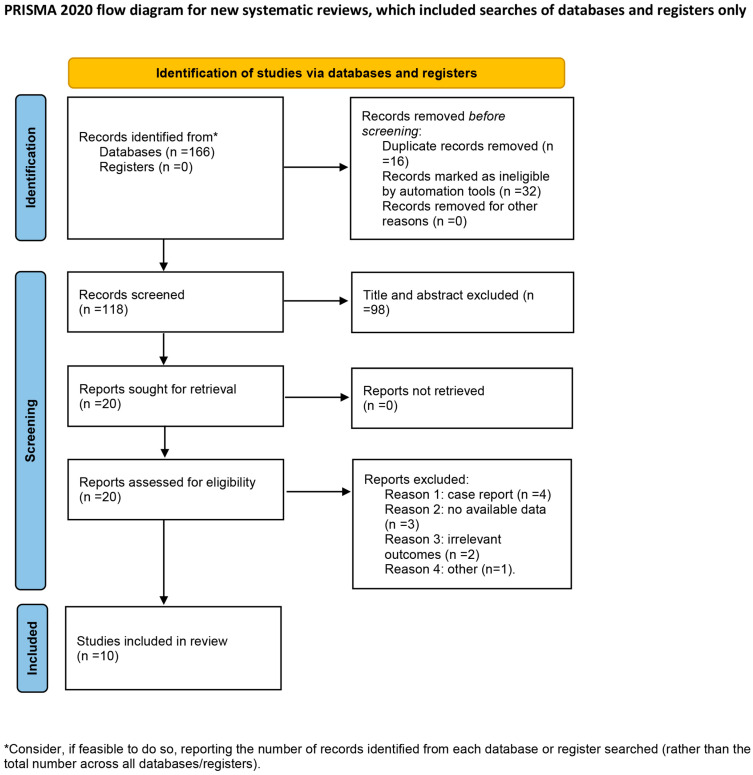
Flow diagram for study selection.

The initial literature search yielded 166 records. Following the removal of duplicates and the screening of titles and abstracts, 20 full-text articles were assessed for eligibility (Figure 1). Ten randomized controlled trials (RCTs), which enrolled a total of 386 participants, met the inclusion criteria [11,14,15,16,17,18,19,20,21,22]. The details of the included RCTs are summarized in Table 1. The studies were conducted in nine countries: the USA (*n* = 3), Spain (*n* = 2), Egypt, Australia, Iran, Ireland, and India (*n* = 1 each). The sample sizes ranged from 8 to 70 participants, with a mean age that spanned 51.8 to 75 years. The intervention characteristics varied across studies: the ultrasound frequency used ranged from 25 kHz to 3 MHz, the treatment duration lasted from four weeks to six months, and the session frequency of UAWD ranged from three times per week to biweekly.

**Figure 2:**
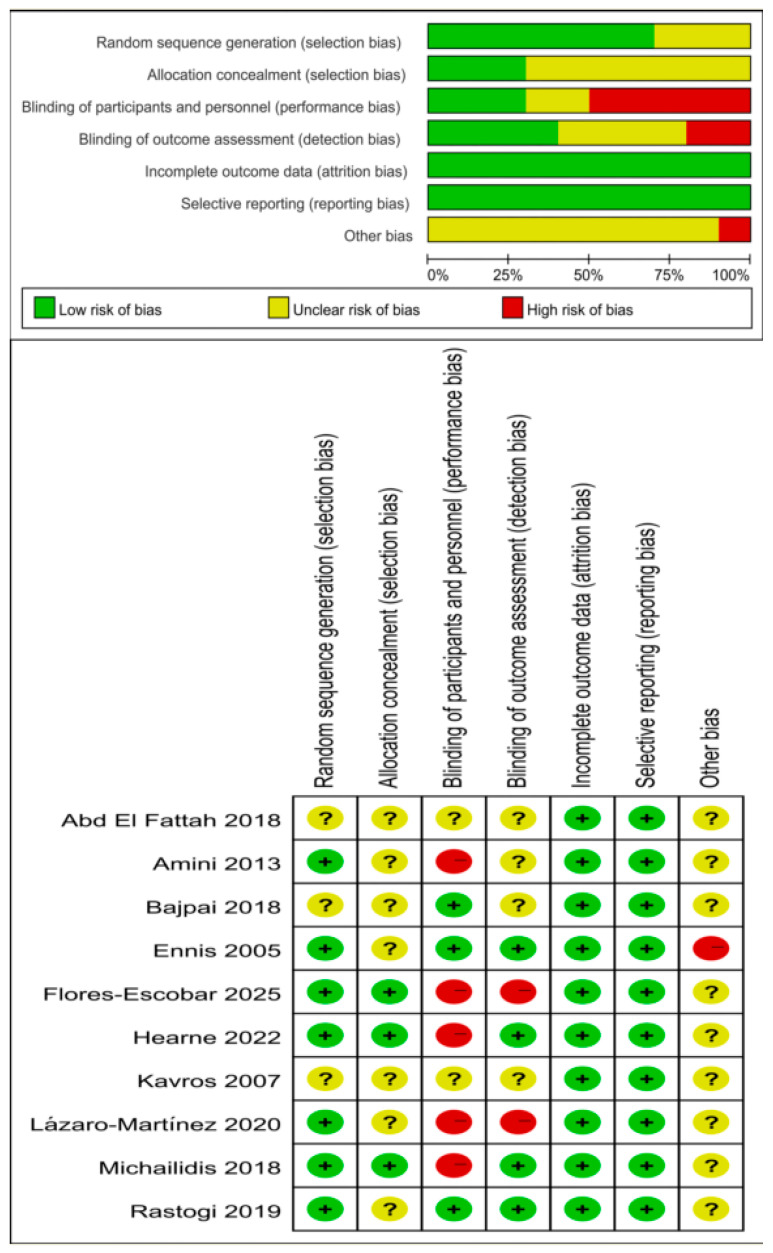
Quality evaluation of the included studies [14,15,16,17,18,19,20,21,22].

The risk of bias assessment of the ten RCTs included is summarized in Figure 2. According to the GRADE system, the overall certainty of evidence for the outcomes of time to healing and wound area reduction percentage was rated as ‘very low’, owing to methodological limitations regarding risk of bias, small sample sizes, substantial heterogeneity, and potential publication bias. In contrast, the certainty of evidence for healing rate was rate as ‘moderate’. A summary of these findings is presented in Table 2.

### 3.2. Meta-Analysis Findings

**Figure 3:**
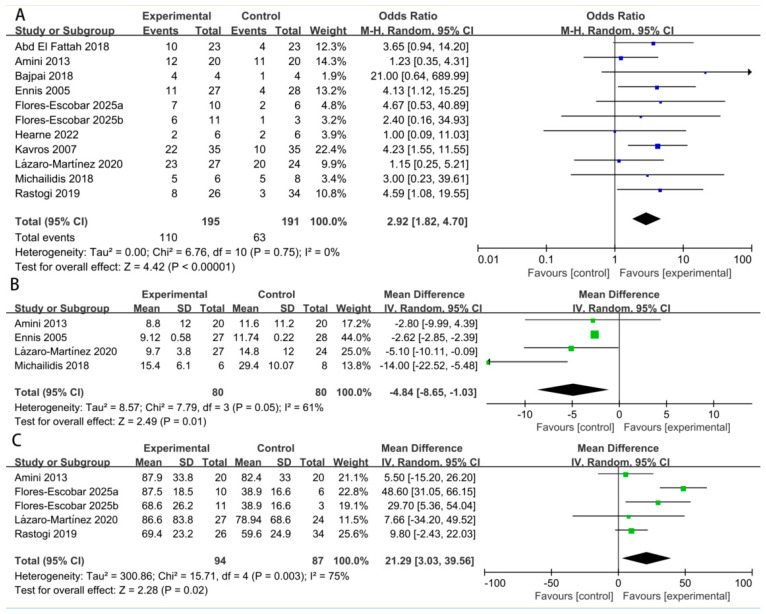
Meta-analysis of healing rate (**A**), time to complete healing (**B**) and wound area reduction percentage (**C**) [14,15,16,17,18,19,20,21,22].

Complete wound healing: Ten studies reported on the complete healing rate. The pooled analysis demonstrated a significant higher wound healing rate when the treatment group was compared with the control group (OR: 2.92; 95% CI: 1.82 to 4.70; *p* = 0.75; I^2^ = 0%) (Figure 3A). The comparison of fixed-effect models and random-effects analyses showed no substantive differences in the direction or significance of the findings. Sensitivity analysis using risk ratios yielded consistent findings (RR: 1.67, 95% CI: 1.16 to 2.41; *p* = 0.01; I^2^ = 57%), confirming the robustness of the results (Table 3).

Time to complete healing: Four studies reported on time to healing. The pooled result indicated that healing was achieved significantly faster in the treatment group by an average of 4.84 weeks (MD: −4.84 weeks; 95% CI: −8.65 to −1.03; *p* = 0.05; I^2^ = 61%) (Figure 3B).

Wound area reduction percentage: Four studies reported quantitative data on the wound area reduction percentage. The meta-analysis showed a significantly greater reduction in wound size in the treatment group (MD: 21.29%; 95% CI: 3.03 to 39.56; *p* = 0.003; I^2^ = 75%) (Figure 3C). Given the variability in measurement time points (ranging from 4 weeks to 6 months) and baseline wound sizes across studies, we performed an additional meta-analysis using standardized mean differences (SMDs) to account for differences in measurement scales. The overall effect remained consistent with the primary analysis using raw mean differences (SMD: 0.58; 95% CI: 0.01 to 1.16; *p* = 0.02; I^2^ = 65%).

### 3.3. Adverse Events

Of the ten studies, six did not provide any safety data, while two only stated that ‘no serious adverse events occurred’ without further details. Ennis et al. documented pain in four cases—three in the control group and one in the ultrasound group—as well as two cases of wound infection in the ultrasound group. Michailidis et al. reported that three cases developed mild soft tissue infections.

### 3.4. Sensitivity Analysis

**Figure 4:**
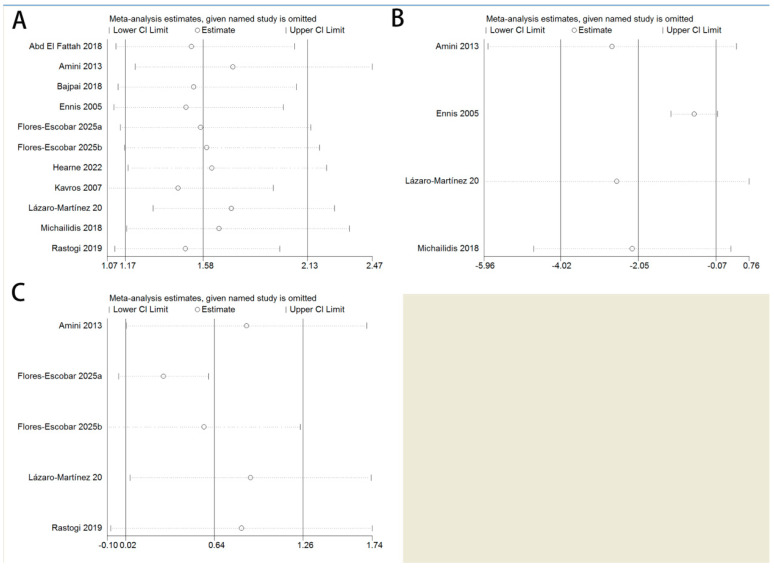
Sensitivity analysis of the meta-analysis using leave-one-out method: (**A**) healing rate, (**B**) time to complete healing, and (**C**) wound area reduction percentage [14,15,16,17,18,19,20,21,22].

We further performed sensitivity analyses to detect whether one trial significantly influenced the outcomes or greatly contributed to the heterogeneity. The leave-one-out analysis confirmed that no single study unduly influenced the overall effect sizes (Figure 4).

### 3.5. Publication Bias

**Figure 5:**
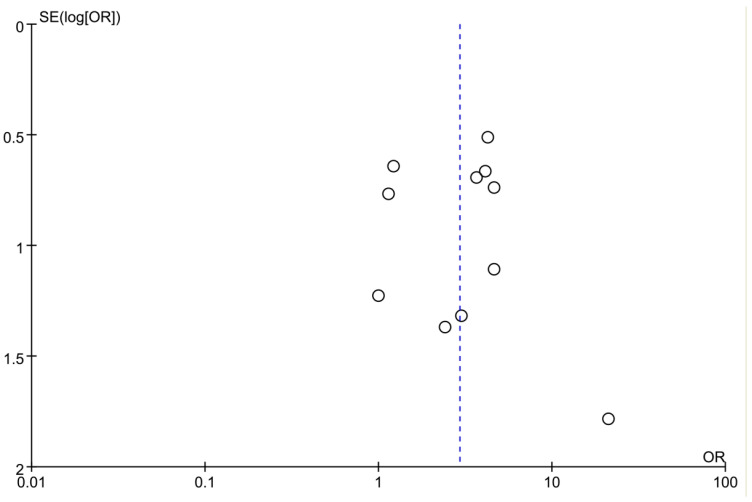
Publication bias analysis of the meta-analysis. Circles = individual studies. Blue dashed vertical line = pooled effect estimate.

Begg’s test and Egger’s test were applied to evaluate the publication bias of healing rate in this study. The funnel plot in the meta-analysis showed no evidence of publication bias in relation to risk of ulcer healing rate (Figure 5). It was also proved by Egger’s test (*p* = 0.149) and Begg’s test (*p* = 1.00). Publication bias was not assessed for the outcomes of time to healing and wound area reduction percentage due to the insufficient number of studies available for each meta-analysis.

## 4. Discussion

In this study, we aimed to conduct a systematic review of the current literature to evaluate the effectiveness of UAWD in DFU management. This meta-analysis provides robust evidence that ultrasound-assisted debridement is superior to conventional debridement methods for treating DFU. The results consistently favored UAWD across all primary efficacy endpoints: a higher proportion of wounds achieved complete closure, a greater percentage reduction in wound size was observed, and healing occurred more rapidly.

The therapeutic benefits of UAWD are likely multifactorial. While its efficacy in removing biofilm and non-viable tissue is well-established, the key differentiator may be its biostimulatory effect [23]. Low-frequency ultrasound is thought to enhance healing at a cellular level by promoting fibroblast proliferation, increasing angiogenesis, and modulating inflammatory cytokines, thereby addressing the underlying pathophysiology of chronic diabetic wounds [24]. Microstreaming refers to the unidirectional flow of interstitial fluids induced by ultrasonic vibrations, which alters cell membrane permeability and modulates second messenger activity. These effects enhance protein synthesis, stimulate mast cell degranulation, and promote the production of growth factors, thereby activating fibroblasts and inducing neoangiogenesis at the wound site [25]. In addition, sharp or surgical debridement necessitates a high level of clinical proficiency, especially when underlying structures such as bone, joint tissue, or ligaments are exposed, given the inherent risk of iatrogenic tissue injury [5]. As a result, UAWD can be a useful alternative to traditional debridement techniques when sharp/surgical debridement is contraindicated, such as in individuals with bad vascular health.

A previous meta-analysis by Flores-Escobar evaluated the effect of UAWD in patients with DFU [26]. The author concluded that UAWD showed a higher healing rate, a greater percentage of wound area reduction, and similar healing times when compared with the control, although no statistically significant differences were observed between groups. Another meta-analysis analyzed the efficacy and safety of UAWD on DFU [27]. In the eleven included articles, five were written in Chinese. There might be a significant bias with low methodological quality. In this study, ten RCTs with 386 patients were included according to the inclusion criteria. The observed outcomes—specifically, an OR of 2.92 for healing rate, a mean reduction of 4.84 weeks in time to wound healing, and a 21.29% greater wound area reduction in the UAWD group—collectively underscore both the clinical relevance of UAWD and its potential to substantially improve the standard of care for patients with DFUs.

The risk of bias assessment revealed substantial methodological concerns across the included studies. Allocation concealment was unclear in seven studies (70%), while blinding of participants and personnel was rated as unclear or at high risk in seven studies (70%), and blinding of outcome assessment was rated as unclear or at high risk in six studies (60%). Inadequate allocation concealment increases the risk of selection bias, potentially leading to systematic baseline differences between groups that favor the intervention. Similarly, lack of blinding in outcome assessment—particularly relevant for wound healing outcomes where subjective judgment is involved—can inflate effect estimates.

These methodological limitations collectively explain the moderate to very low certainty of evidence according to the GRADE framework. The absence of blinding introduces a high risk of performance bias, especially for subjective outcomes such as time to healing, where treatment effects may be overestimated. Additionally, imprecision is a major concern: small sample sizes resulted in wide confidence intervals for the pooled estimates of the time to healing and wound area reduction percentage, directly leading to very low GRADE ratings for these outcomes. Given that most included studies had an unclear or high risk of bias in key domains, the observed treatment effects are likely overestimates of the true effect. Therefore, while our findings suggest that ultrasound-assisted debridement may be effective, the magnitude of benefit remains uncertain, and the true effect may be more modest than reported. Future RCTs should prioritize rigorous methodological quality, including adequate allocation concealment and blinded outcome assessment, to provide more reliable estimates of treatment efficacy.

The significant heterogeneity in treatment parameters across the included studies—such as ultrasound frequency (ranging from 25 kHz to 3 MHz) and treatment frequency (ranging from three times per week to biweekly)—precludes the recommendation of a single, optimized protocol. For example, Flores-Escobar et al. compared weekly versus biweekly UAWD in patients with DFU and found no significant difference in healing rates between the two regimens [14]. Furthermore, an expert panel recommended that UAWD may be performed sequentially, potentially at each dressing change if necessary [25]. This variability in treatment delivery represents a considerable confounding factor that may affect the outcome comparability. Another important limitation is the heterogeneity in outcome definitions across included studies. For the wound area reduction, measurement time points varied from 4 to 24 weeks. For complete healing, definitions of ‘healed’ were generally consistent (100% epithelialization), but the maximum follow-up durations varied from 6 to 24 weeks. Studies with a shorter follow-up may underestimate the proportion of patients that achieved complete healing. Translating these findings into clinical practice faces several challenges, including the need for specialized equipment and trained operators, the absence of standardized protocols due to inconsistent parameters, and the lack of economic data for cost-effectiveness evaluation. Consequently, future guidelines should aim to establish standardized treatment parameters based on robust efficacy and safety evidence.

This systematic review has several limitations. First, the analysis included only ten RCTs with a total of 386 patients. A small number of included RCTs with limited sample sizes and varying follow-up durations across these studies limit the statistical power and generalizability of the findings. Small trials are known to exaggerate treatment effects and increase the risk of publication bias. Although our sensitivity analyses suggested that no single study unduly influenced the pooled estimate, the possibility of small-study effects cannot be entirely excluded. Second, the time to complete healing was pooled using mean differences rather than hazard ratios due to insufficient reporting in the primary studies. This approach does not account for censoring and may underestimate or overestimate treatment effects. Third, significant heterogeneity was observed for the outcomes of time to healing (I^2^ = 61%) and wound area reduction percentage (I^2^ = 75%), which limits the certainty and generalizability of their clinical interpretation. Formal subgroup analyses to investigate sources of heterogeneity were not feasible due to the limited number of studies; however, a leave-one-out sensitivity analysis confirmed the directional robustness of the pooled estimates. Fourth, critical reporting gaps constrain the interpretation: inadequate reporting of patient-centered outcomes (e.g., quality of life, pain, and cost-effectiveness) precluded a comprehensive assessment of their broader clinical implications, while sparse data limit the evaluation of long-term outcomes (e.g., recurrence, amputation). Fifth, the safety profile of the intervention could not be adequately characterized due to the incomplete reporting of adverse events in the included trials, which precludes a comprehensive risk–benefit assessment. Sixth, although we conducted a comprehensive search across multiple databases, restricting the search to English-language publications may have introduced language bias, potentially excluding relevant studies published in other languages. Additionally, the variability in control interventions reflects real-world clinical practice but also limits the precision of our effect estimates. Future studies should adopt standardized protocols for control groups or clearly document all components of standard care to facilitate comparisons across trials. Finally, publication bias assessment was constrained by the limited number of studies. While a funnel plot inspection and Egger’s test did not show significant asymmetry, these methods have low power with fewer than 10 studies and should be interpreted cautiously. To address the limitations identified and build upon our findings, future research should prioritize the following: (1) large, pragmatic RCTs with adequate sample size; (2) standardized intervention protocols that clearly define and justify ultrasound parameters, enabling reproducibility and pooled analysis; and (3) adoption of a core outcome set for DFU trials, mandating consistent reporting of healing metrics, patient-reported outcomes (e.g., pain, quality of life), adverse events, and long-term endpoints like ulcer recurrence.

## 5. Conclusions

This meta-analysis demonstrates that UAWD is a highly effective and safe therapeutic modality that significantly improves healing outcomes for diabetic foot ulcers compared with conventional debridement alone. It could be considered as a potential adjunctive option within standardized wound care pathways. However, these findings are tempered by several important limitations. The certainty of evidence was low to very low for most outcomes, substantial heterogeneity was observed across studies, and adverse event reporting was incomplete. Without robust safety data, the true risk–benefit profile of this intervention cannot be adequately assessed, and widespread clinical adoption cannot yet be recommended. Further high-quality, adequately powered randomized controlled trials with standardized outcome reporting are needed to confirm these findings.

## Figures and Tables

**Table 1:** The characteristics of the included studies.

	Country	Study Design	Ulcer Grade	No.		Intervention		Mean Age (Years)	Ultrasound Frequency	Outcome	Adverse Event	Treatment Time
				TG	CG	TG	CG					
Abd El Fattah 2018 [22]	Egypt	RCT	NR	23	23	SC + UAWD	SC	NR	NR	1	NR	12 weeks
Amini 2013 [21]	Iran	RCT	Wagner Classification: 3	20	20	SC + UAWD	SC	55.2	NR	1, 2, 3	NR	6 months
Bajpai 2018 [15]	USA	RCT	NR	4	4	SC + UAWD (weekly)	SC	57.6	25 kHz	1	NR	12 weeks
Ennis 2005 [16]	USA	RCT	Wagner Classifications: 1, 2	27	28	SC + UAWD (weekly)	SC	NR	40 kHz	1, 2	Pain: 4, Infection: 2	12 weeks
Flores-Escobar 2025 [14]	Spain	RCT	Texas Classifications: 1, 2	10	9	SC + UAWD (weekly)	SC	70.2	25 kHz	1, 3	NR	12 weeks
				11		SC + UAWD (biweekly)						
Hearne 2022 [11]	Ireland	RCT	NR	6	5	SC + UAWD (twice a week)	SC	71.6	1.0–3.0 MHz	1	0	8 weeks
Kavros 2007 [17]	USA	RCT	NR	35	35	SC + UAWD (3 times a week)	SC	75	40 kHz	1	NR	12 weeks
Lázaro-Martínez 2020 [18]	Spain	RCT	Texas Classifications: 1, 2	27	24	SC + UAWD (weekly)	SC	61.2	25 kHz	1, 2, 3	NR	6 weeks
Michailidis 2018 [19]	Australia	RCT	Texas Classifications: 1, 2	6	8	SC + UAWD (weekly)	SC	NR	NR	1, 2	Infection: 3	6 weeks
Rastogi 2019 [20]	India	RCT	Wagner Classifications: 2, 3	26	34	SC + UAWD (daily for the initial 6 days, followed by twice a week)	SC	51.8	26 kHz to 60 kHz	1, 3	NR	4 weeks

TG: treatment group; CG: control group; SC: standard of care treatment; NR: not reported; 1: healing rate, 2: time to complete healing, 3: wound area reduction percentage.

**Table 2:** Grading of Recommendations Assessment, Development and Evaluation (GRADE) assessment of overall certainty.

Outcomes	No. of Studies	Risk of Bias	Inconsistency	Indirectness	Imprecision	Publication Bias	Quality of Evidence
Healing rate	10	Serious	Not serious	Not serious	Not serious	Not serious	⨁⨁⨁◯ Moderate
Time to healing	4	Serious	Serious	Not serious	Serious	Serious	⨁◯◯◯ Very Low
Wound area reduction percentage	4	Serious	Serious	Not serious	Serious	Serious	⨁◯◯◯ Very Low

The GRADE system rates evidence as Moderate (⨁⨁⨁◯), or Very low (⨁◯◯◯), where each ⨁ represents one point and ◯ represents zero points.

**Table 3:** Sensitive analysis for outcome of healing rate.

	OR				RR			
	Effect Size	95% CI	*p*	I^2^	Effect Size	95% CI	*p*	I^2^
Fixed-effect models	2.94	1.85–4.68	0.75	0	1.64	1.32–2.04	0.01	57%
Random-effects models	2.92	1.82–4.70	0.75	0	1.67	1.16–2.41	0.01	57%

## Data Availability

The original contributions presented in this study are included in the article/Supplementary Material. Further inquiries can be directed to the corresponding authors.

## Supplementary Materials

The following supporting information can be downloaded from https://www.mdpi.com/article/10.3390/biomedicines14040846/s1, File S1: Search strategy.
