# Supplementary material for: Ultrasound-Assisted Wound Debridement for Diabetic Foot Ulcers: A Systematic Review and Meta-Analysis of Randomized Controlled Trials
Source: Biomedicines. 2026 Apr 8;14(4):846. doi: 10.3390/biomedicines14040846 (PMC13113055; doi:10.3390/biomedicines14040846)
Supplement: Supplementary file 1 [file biomedicines-14-00846-s001.zip › biomedicines-4191654-supplementary.pdf]

Pubmed

No.	Query
#1	"diabetic foot"[Mesh] OR "diabetic foot"[tiab] OR "diabetic feet"[tiab] OR "diabetic foot ulcer"[tiab] OR "DFU"[tiab] OR "foot ulcer"[tiab] OR "diabetes complications"[Mesh]
#2	"ulcer"[Mesh] OR "ulcer"[tiab] OR "ulcers"[tiab] OR "ulceration"[tiab] OR "wound"[tiab] OR "wounds"[tiab]
#3	#1 AND #2
#4	((Ultrasound-Assisted Wound Debridement) OR (ultrasonic debridement)) OR (ultrasound debridement) OR (sonic debridement)
#5	#3 AND #4

Embase

No.	Query
#1	'diabetic foot'/exp OR 'diabetic foot':ti,ab OR 'diabetic feet':ti,ab OR 'diabetic foot ulcer':ti,ab OR 'dfu':ti,ab OR 'foot ulcer'/exp
#2	'ulcer'/exp OR 'ulcer':ti,ab OR 'ulcers':ti,ab OR 'ulceration':ti,ab OR 'wound'/exp OR 'wound':ti,ab
#3	#1 AND #2
#4	'Ultrasound-Assisted Wound Debridement':ab,ti OR 'ultrasonic debridement':ab,ti OR 'ultrasound debridement':ab,ti
#5	#3 AND #4

Web of science

No.	Query
#1	TS=("diabetic foot" OR "diabetic feet" OR "diabetic foot ulcer" OR "DFU" OR "foot ulcer")
#2	TS=("ulcer" OR "ulcers" OR "ulceration" OR "wound" OR "wounds")
#3	#1 AND #2
#4	TS=("Ultrasound-Assisted Wound Debridement" OR "ultrasound debridement" OR "ultrasonic debridement" OR "sonic debridement")
#5	#3 AND #4

BIOSIS

No.	Query
#1	TS=("diabetic foot" OR "diabetic foot ulcer" OR "DFU")
#2	TS=("ulcer″ OR "wound")
#3	#1 AND #2
#4	TS=("Ultrasound-Assisted Wound Debridement" OR "ultrasound debridement" OR "ultrasonic debridement" OR "sonic debridement")
#5	#3 AND #4
